# Description and Genomic Characteristics of *Weissella fermenti* sp. nov., Isolated from Kimchi

**DOI:** 10.4014/jmb.2306.06010

**Published:** 2023-07-25

**Authors:** Jae Kyeong Lee, Ju Hye Baek, Dong Min Han, Se Hee Lee, So Young Kim, Che Ok Jeon

**Affiliations:** 1 Department of Life Science, Chung-Ang University, Seoul 06974, Republic of Korea; 2 Microbiology and Functionality Research Group, World Institute of Kimchi, Gwangju, 61755, Republic of Korea; 3 Department of Agro-Food Resources, National Institute of Agricultural Sciences, Rural Development Administration, Wanju 55365, Republic of Korea

**Keywords:** *Weissella fermenti*, new taxa, taxonomy, kimchi, genome

## Abstract

A Gram-positive, non-motile, and non-spore-forming lactic acid bacterium, designated as BK2^T^, was isolated from kimchi, a Korean traditional fermented vegetable food, and the taxonomic characteristics of strain BK2^T^, along with strain LMG 11983, were analyzed. Both strains optimally grew at 30°C, pH 7.0, and 1.0% NaCl. Cells of both strains were heterofermentative and facultatively anaerobic rods, demonstrating negative reactions for catalase and oxidase. Major fatty acids (>10%) identified in both strains were C_18:1_ *ω*9*c*, C_16:0_, and summed feature 7 (comprising C_19:1_ *ω*6*c* and/or C_19:1_ *ω*7*c*). The genomic DNA G+C contents of both strains were 44.7 mol%. The 16S rRNA gene sequence similarity (99.9%), average nucleotide identity (ANI; 99.9%), and digital DNA-DNA hybridization (dDDH; 99.7%) value between strains BK2^T^ and LMG 11983 indicated that they are different strains of the same species. Strain BK2^T^ was most closely related to *Weissella confusa* JCM 1093^T^ and *Weissella cibaria* LMG 17699^T^, with 100% and 99.4% 16S rRNA gene sequence similarities, respectively. However, based on the ANI and dDDH values (92.3% and 48.1% with *W. confusa*, and 78.4% and 23.5% with *W. cibaria*), it was evident that strain BK2^T^ represents a distinct species separate from *W. confusa* and *W. cibaria*. Based on phylogenetic, phenotypic, and chemotaxonomic features, strains BK2^T^ and LMG 11983 represent a novel species of the genus *Weissella*, for which the name *Weissella fermenti* sp. nov. is proposed. The type of strain is BK2^T^ (=KACC 22833^T^=JCM 35750^T^).

## Introduction

Since the genus *Weissella* was first proposed with *Weissella viridescens* as a member of the family *Leuconostocaceae* in 1993 [1], many new *Weissella* species have been proposed, and certain *Weissella* species have been reclassified either as synonyms or as members of a newly established genus *Periweissella* [2]. At the time of writing, the genus *Weissella* encompasses 20 validly and 2 invalidly published species (https://lpsn.dsmz.de/genus/weissella), which have primarily been isolated from various foods, including fermented sausage [1], kimchi [3], yogurt [4], fermented fish [5], fermented rice [6, 7], and sugar cane [8]. However, many *Weissella* members have also been isolated from a wide range of other diverse habitats, such as feces or gut of animals and insects, clinical samples, fish, soil, and waters [9-15]. Members of the genus *Weissella* are typically characterized as Gram-positive, facultatively anaerobic, catalase- and oxidase-negative, non-motile, with a relatively low DNA G+C (37–47 mol%) content, and heterofermentative lactic acid bacteria producing D- or DL-lactic acids depending on species, and their cells appear as short rods with rounded tapered ends or ovoid shapes, often occurring in pairs or short chains [9, 16].

Many *Weissella* species, including *Weissella cibaria*, *Weissella confusa*, *Weissella koreensis*, and *Weissella paramesenteroides*, have been reported to be associated with various health benefits and functionalities, such as atopic disease improvement, anti-inflammatory effects, and anti-obesity effects [17-20]. Due to these characteristics, *Weissella* species have been considered as starter cultures for food fermentation processes to improve food quality and functionality, and as potential probiotics to promote health [21]. In this study, we isolated a putative novel *Weissella* strain, designated as BK2^T^, from kimchi, a Korean traditional fermented vegetable food, and subsequently analyzed the taxonomic characteristics of strain BK2^T^, along with strain LMG 11983 isolated from grass silage, using a polyphasic approach.

## Material and Methods

### Isolation and Cultivation

Strain BK2^T^ was isolated from fermented baechu kimchi prepared using baechu cabbage (*Brassica rapa* subsp. *pekinensis*) as a major raw material according to the method described previously [22] in South Korea. A small amount of supernatant obtained from baechu kimchi, which was fermented for 20 days at 4°C, was serially diluted in 0.9% (w/v) saline, spread on de Man, Rogosa, and Sharpe (MRS, USA) agar, and incubated at 30°C for 2 days. The crude genomic DNA (gDNA) was extracted from colony cells grown on MRS agar by subjecting them to boiling in 100 μl of a 5% solution of Chelex 100 (Bio-Rad, USA) for 10 min. Subsequently, the 16S rRNA genes within the crude gDNA were amplified by PCR using the universal primers F1 (5'-AGA GTT TGA TCM TGG CTC AG-3') and R13 (5'-TAC GGY TAC CTT GTT ACG ACT T-3'), and the resulting PCR products were then subjected to double digestion with the restriction enzymes HaeIII and HhaI [23]. The PCR amplicons, displaying distinctive digestion patterns, were partially sequenced using the primer 340F (5'-CCT ACG GGA GGC AGC AG-3') [23] at Macrogen (Korea). Subsequently, the obtained 16S rRNA gene sequences were compared with the 16S rRNA gene sequences of all validly and invalidly published bacterial type strains using the Nucleotide Similarity Search program available on the EzBioCloud server (www.ezbiocloud.net/identify) [24]. From the comparison, a *Weissella* strain, designated as BK2^T^, was selected for phenotypic and phylogenetic analyses. Strain BK2^T^ was routinely cultured on MRS agar for 2 days at 30°C. For long-term preservation, the strain was stored at –80°C in MRS broth supplemented with 15% (v/v) glycerol. Strain LMG 11983, *W. confusa* KACC 11841^T^, *W. cibaria* KACC 11862^T^, *Weissella muntiaci* NBRC 113537^T^, and *W. viridescens* KACC 11850^T^ were obtained from their culture collection centers and used as reference strains for the comparison of phenotypic and genomic properties and fatty acid compositions.

### Phylogenetic Analysis Based on 16S rRNA Gene Sequences

To obtain nearly complete sequences of the 16S rRNA genes of strains BK2^T^ and LMG 11983, PCR amplification was performed using the F1 and R13 primers. The resulting PCR amplicons were subjected to sequencing using the 340F, 518R (5'-ATT ACC GCG GCT GCT GG-3'), and 805F (5'-GAT TAG ATA CCC TGG TAG TC-3') primers, and the resulting nucleotide sequences were assembled as described previously [23]. The 16S rRNA gene sequence similarities between strains BK2^T^ and LMG 11983 and other bacterial type strains were calculated using the EzBioCloud server. The 16S rRNA gene sequences of strains BK2^T^ and LMG 11983 and closely related valid type strains were aligned using the secondary-structure aware Infernal aligner [25]. Phylogenetic trees were constructed using the neighbor-joining (NJ), maximum-likelihood (ML), and maximum-parsimony (MP) algorithms with bootstrap values (1,000 replications) in MEGA11 [26]. The Kimura two-parameter model, nearest-neighbor-interchange heuristic search method, and complete deletion options were used for the NJ, ML, and MP tree constructions, respectively.

### Genome Sequencing and Phylogenomic Analysis

The gDNA of strains BK2^T^ and LMG 11983 was extracted using a Wizard Genomic DNA Purification Kit (Promega, USA) following the manufacturer's instructions. The gDNA of strain BK2^T^ was sequenced using a hybrid combination of the Oxford Nanopore MinION platform (Nanopore, UK) in our laboratory and the Illumina HiSeq X platform (Illumina, USA) with 151 bp paired-end sequencing reads at Macrogen. On the other hand, the gDNA of strain LMG 11983 was sequenced using the Oxford Nanopore MinION platform. Sequencing reads derived from the Nanopore MinION sequencing were de novo-assembled using Flye ver. 2.9.1 [27]. The assembled long contigs of strain BK2^T^ were polished with Illumina sequencing reads using Pilon ver. 1.24 [28], and multiple rounds of polishing were performed until no additional corrections were accomplished. The quality of the assembled genomes was assessed by evaluating their completeness and contamination rates using CheckM ver. 1.0.4 [29]. Finally, the genome sequences of strains BK2^T^ and LMG 11983 were submitted to GenBank and annotated using the NCBI Prokaryotic Genome Annotation Pipeline (PGAP; www.ncbi.nlm.nih.gov/genome/annotation_prok/) [30].

The DNA G+C contents of the strains were determined based on their whole genome sequences. To perform genome-based phylogenomic analysis, 92 housekeeping core genes were extracted from the genomes of strains BK2^T^ and LMG 11983, as well as from closely related *Weissella* type strains, using the Up-to-date Bacterial Core Gene (UBCG) pipeline (https://help.ezbiocloud.net/ubcg-gene-set/) [31]. Subsequently, a phylogenomic tree was constructed using the ML algorithm in MEGA 11, based on the amino acid sequences of the core genes, with bootstrap values (1,000 replications). Average nucleotide identity (ANI) and digital DNA-DNA hybridization (dDDH) value were calculated to assess the relatedness among strains BK2^T^ and LMG 11983, as well as reference strains *W. confusa* NRBC 106469^T^, *W. cibaria* JCM 12495^T^, *W. muntiaci* 8H-2^T^ and *W. viridescens* DSM 20410^T^ using the Orthologous Average Nucleotide Identity Tool (OAT) software, available on the EzBioCloud server (www.ezbiocloud.net/sw/oat) [32], and the Genome-to-Genome Distance Calculator ver. 2.1 (http://ggdc.dsmz.de/distcalc2.php) [33], a web-based tool, respectively. The general genomic features of strains BK2^T^ and LMG 11983 and closely related type strains of the genus *Weissella* were obtained from GenBank annotated by the NCBI PGAP. Protein-coding genes derived from the genomes of strains BK2^T^ and LMG 11983 and reference strains were categorized using the Clusters of Orthologous Genes (COG) database based on sequence similarities computed using the USEARCH program (ver. 9.0) with default parameter settings [34].

### Phenotypic, Physiological, and Biochemical Analyses

Strains BK2^T^ and LMG 11983 were tested for their growth on various culture media including MRS agar, Reasoner's 2A (R2A) agar (BD), tryptic soy agar (TSA; BD), nutrient agar (NA; BD), marine agar (MA; BD), and Luria-Bertani (LB) agar (BD) for 2 days at 30°C. To evaluate the growth temperature and pH ranges of strains BK2^T^ and LMG 11983, they were incubated on MRS agar and in MRS broth at different temperatures (10–50°C at 5°C intervals) and pH values (4.0–10.0 at 1.0 pH unit intervals), respectively, for 2 days. MRS broths with pH 4.0–5.0, 6.0–8.0, and 9.0–10.0 were prepared using sodium citrate, Na_2_HPO_4_/NaH_2_PO_4_, and Tris-HCl buffers, respectively [35]. The pH values were adjusted if necessary after autoclaving at 121°C for 15 min. The MRS broths used for pH testing were incubated without agitation at 30°C. The growth of strains BK2^T^ and LMG 11983 was also examined in MRS broth containing different concentrations of NaCl (0–5%, w/v, at 1.0% intervals), prepared in the laboratory according to the MRS formula.

The biochemical and physiological properties of strains BK2^T^ and LMG 11983 were evaluated using cells grown on MRS agar for 2 days at 30°C. Gram staining was performed using PREVI Colour Gram (bioMérieux, France), following the manufacturer's instructions. The cell morphology of strains BK2^T^ and LMG 11983 was examined using phase-contrast microscopy (Carl Zeiss, Germany) and transmission electron microscopy (JEM-1010; Jeol, Japan). Oxidase and catalase activities were determined by observing the oxidation of 1% (w/v) tetramethyl-*p*-phenylenediamine (Merck, USA) and the production of oxygen bubbles in a 3% (v/v) aqueous hydrogen peroxide solution, respectively.

The phenotypic characteristics of strains BK2^T^ and LMG 11983, along with four closely related reference strains, were examined under identical growth conditions. Hydrolysis of tyrosine, casein, esculin, gelatin, starch, Tween 20, and Tween 80 was assessed on MRS agar following previously described protocols [36, 37]. Additional biochemical features and enzymatic activities were determined using the API 20E and API ZYM systems (bioMérieux), respectively, according to the manufacturer's instructions. Acid production from various carbohydrates was evaluated using API 50 CH test strips (bioMérieux) with the API 50 CHL medium, following the manufacturer's guidelines. The configuration of lactic acids produced by strains BK2^T^ and LMG 11983 and reference strains was determined using a DL-lactate test kit (Boehringer Mannheim/R-Biopharm, Germany).

### Chemotaxonomic Analyses

For cellular fatty acid analysis, strains BK2^T^ and LMG 11983, along with four reference strains, were cultivated in MRS broth at 30°C. Microbial cells were harvested during the exponential growth phase at an 0.8 optical density at 600 nm. The cellular fatty acids of the harvested cells were saponified, methylated, and extracted following the standard Sherlock Microbial Identification System (MIDI) protocol. Fatty acid methyl esters were analyzed by gas chromatography on the HP 6890 GC (Hewlett Packard, USA) and identified using the MIDI identification database RTSBA6 (ver. 6.0B). Polar lipids of strains BK2^T^ and LMG 11983 were extracted from cells harvested during the exponential growth phase and analyzed by two-dimensional thin-layer chromatography following the protocol described by Minnikin *et al*. [38]. Various reagents were used to identify different types of polar lipids, including 10% ethanolic molybdophosphoric acid for total polar lipids, ninhydrin for aminolipids, Dittmer-Lester reagent for phospholipids, and *α*-naphthol/sulfuric acid for glycolipids. To confirm the presence or absence of phosphatidylglycerol (PG) and diphosphatidylglycerol (DPG) in strains BK2^T^ and LMG 11983, standard compounds of PG and DPG obtained from Sigma-Aldrich (USA) were employed.

## Results and Discussion

### Phylogenetic Analysis Based on 16S rRNA Gene Sequences

Sequencing using the 340F, 518R, and 805F primers yielded nearly complete 16S rRNA gene sequences of strains BK2^T^ (1,513 nucleotides) and LMG 11983 (1,504 nucleotides). The obtained sequences of strains BK2^T^ and LMG 11983 were found to be very similar, showing a 99.9% sequence similarity, indicating their close phylogenetic relatedness. Comparative analysis of the 16S rRNA gene sequences revealed that strain BK2^T^ exhibited the highest similarities to *W. confusa* JCM 1093^T^ and *W. cibaria* LMG 17699^T^, with 100% and 99.4% sequence similarities, respectively. The 16S rRNA gene sequence similarities with other bacterial type strains were below 97.4%, which falls below the recommended threshold value for distinguishing different species based on 16S rRNA gene similarity [39], indicating that strains BK2^T^ and LMG 11983 represent distinct species separate from the other species except for *W. confusa* and *W. cibaria*.

**Fig. 1:**
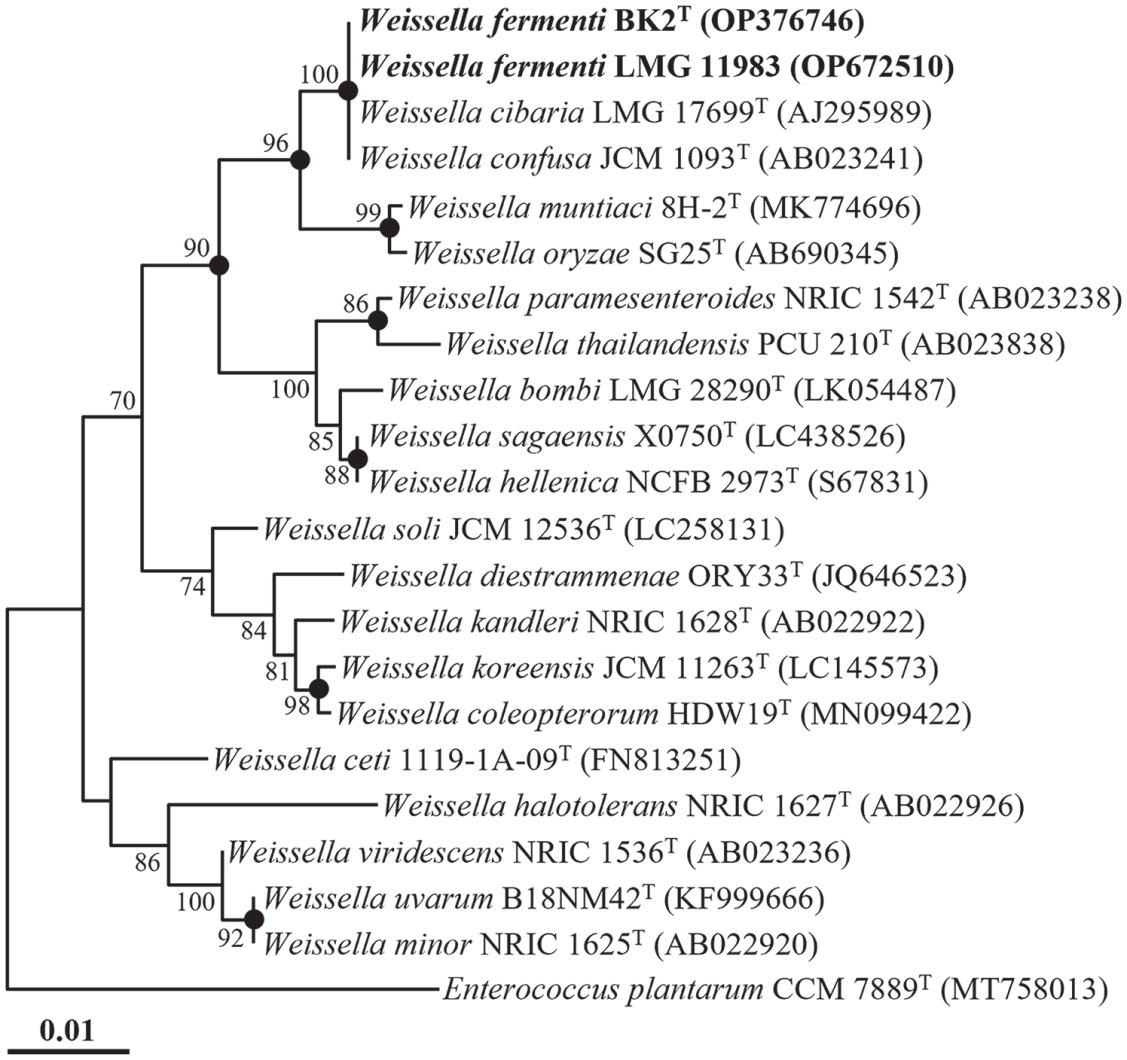
A neighbor-joining tree (NJ) showing the phylogenetic relationships between strains BK2^T^ and LMG 11983 and closely related species based on 16S rRNA gene sequences. Bootstrap values (based on 1,000 replications) exceeding 70% are indicated at branch points. The filled circles (●) indicate branches that were commonly recovered in NJ, maximum-likelihood, and maximum-parsimony algorithms. *Enterococcus plantarum* CCM 7889^T^ (MT758013) was used as the outgroup. The scale bar equals 0.01 changes per nucleotide position.

Phylogenetic analysis using the NJ algorithm based on the 16S rRNA gene sequences demonstrated that strains BK2^T^ and LMG 11983 formed a tight phyletic lineage with *W. confusa* JCM 1093^T^ and *W. cibaria* LMG 17699^T^ within the genus *Weissella*, with a robust bootstrap value of 100% (Fig. 1). This result was consistent with the phylogenetic trees generated using the ML and MP algorithms (Fig. S1). Based on the sequence similarity and phylogenetic analyses of the 16S rRNA gene sequences, it is suggested that strains BK2^T^ and LMG 11983 can be classified as members of the genus *Weissella*.

### Genomic Features and Phylogenomic and Genome Relatedness

The *de novo* assembly of the genome sequencing data for strains BK2^T^ and LMG 11983 resulted in draft genomes of approximately 2,497.6 and 2,507.5 kb in size, consisting of 5 and 2 contigs, respectively. The 16S rRNA gene sequences identified in the genomes of strains BK2^T^ and LMG 11983 exhibited nearly identical matches to those obtained from their corresponding PCR products, indicating accurate genome sequencing of strains BK2^T^ and LMG 11983. The completeness and contamination rates of the genomes were determined to be 99.1% and 0%for strain BK2^T^ and 98.9% and 0% for strain LMG 11983, respectively, satisfying the criteria (≥ 90% completeness and ≤10% contamination) for high-quality genomes [29]. Detailed sequencing summaries and general genomic features of strains BK2^T^ and LMG 11983, along with comparisons to closely related type strains of the genus *Weissella*, are presented in Table 1. The DNA G+C contents of strains BK2^T^ and LMG 11983, calculated from their whole genomes, were both determined to be 44.7 mol%, which falls within the range of DNA G+C contents observed in *Weissella* species [9]. The DNA G+C contents of strains BK2^T^ and LMG 11983 were found to be similar to those of their closely related *Weissella* species, *W. confusa* and *W. cibaria*, but these values were noticeably higher than those observed in other reference *Weissella* species, *W. muntiaci* 8H-2^T^ and *W. viridescens* DSM 20410^T^. Notably, strains BK2^T^ and LMG 11983 have larger genome sizes (2.50–2.51 Mb) than other closely related *Weissella* species. Correspondingly, these strains harbor a greater number of genes compared to other closely related *Weissella* species. In particular, strains BK2^T^ and LMG 11983 have considerably larger genomes and more genes (2,433–2,506 genes) than *W. viridescens* (1.54 Mb, 1,546 genes), the type species of the genus *Weissella*. Despite strains BK2^T^ and LMG 11983 having larger genomes and more protein-coding genes, the numbers of protein-coding genes assigned to COG in these strains were similar to those of *W. confusa* and *W. cibaria* (Table 1), which indicates that strains BK2^T^ and LMG 11983 harbor more protein-coding genes that are not assigned to COG categories than *W. confusa* and *W. cibaria*. However, these unclassified protein-coding genes may also contribute to the enhanced adaptability of strains BK2^T^ and LMG 11983 to diverse environments compared to *W. confusa* and *W. cibaria*. In addition, strains BK2^T^ and LMG 11983 contain a distinctly higher number of rRNA and tRNA genes, potentially indicative of their higher metabolic activities, compared to other *Weissella* species.

Clustered regularly interspaced short palindromic repeats (CRISPRs) have been widely recognized as a host defense mechanism against bacteriophage predation [40]. Interestingly, the genomes of strains BK2^T^ and LMG 11983 were found to harbor a single CRISPR region, unlike other *Weissella* species strains such as *W. confusa* and *W. cibaria*, which lack this feature (Table 1). This observation strongly suggests that strains BK2^T^ and LMG 11983 have encountered bacteriophage infections in their evolutionary history [41]. These findings suggest that compared to other *Weissella* species, strains BK2^T^ and LMG 11983 may possess a more diverse lifestyle and exhibit complicated regulatory systems that enable them to respond more elaborately to environmental changes.

Strain BK2^T^ was found to possess three distinct types of the lactate dehydrogenase gene (*ldh*). These included individual copies of the NADH-dependent L-*ldh* (E.C. 1.1.1.27) and NADH-dependent D-*ldh* (E.C. 1.1.1.28) genes, as well as one copy of a quinol-dependent membrane-bound D-*ldh* (E.C. 1.1.5.12) gene. This *ldh* gene composition is in common with *W. confusa* NBRC 106469^T^ and different from *W. cibaria* JCM 12495^T^, which harbors two copies of the L-*ldh* gene and one copy of the D-*ldh* gene but lacks a membrane-bound D-*ldh* gene, and *W. muntiaci* 8H-2^T^, which carries only one copy of the D-*ldh* gene, suggesting the capability of *W. muntiaci* 8H-2^T^ to predominantly produce D-lactic acid.

**Fig. 2:**
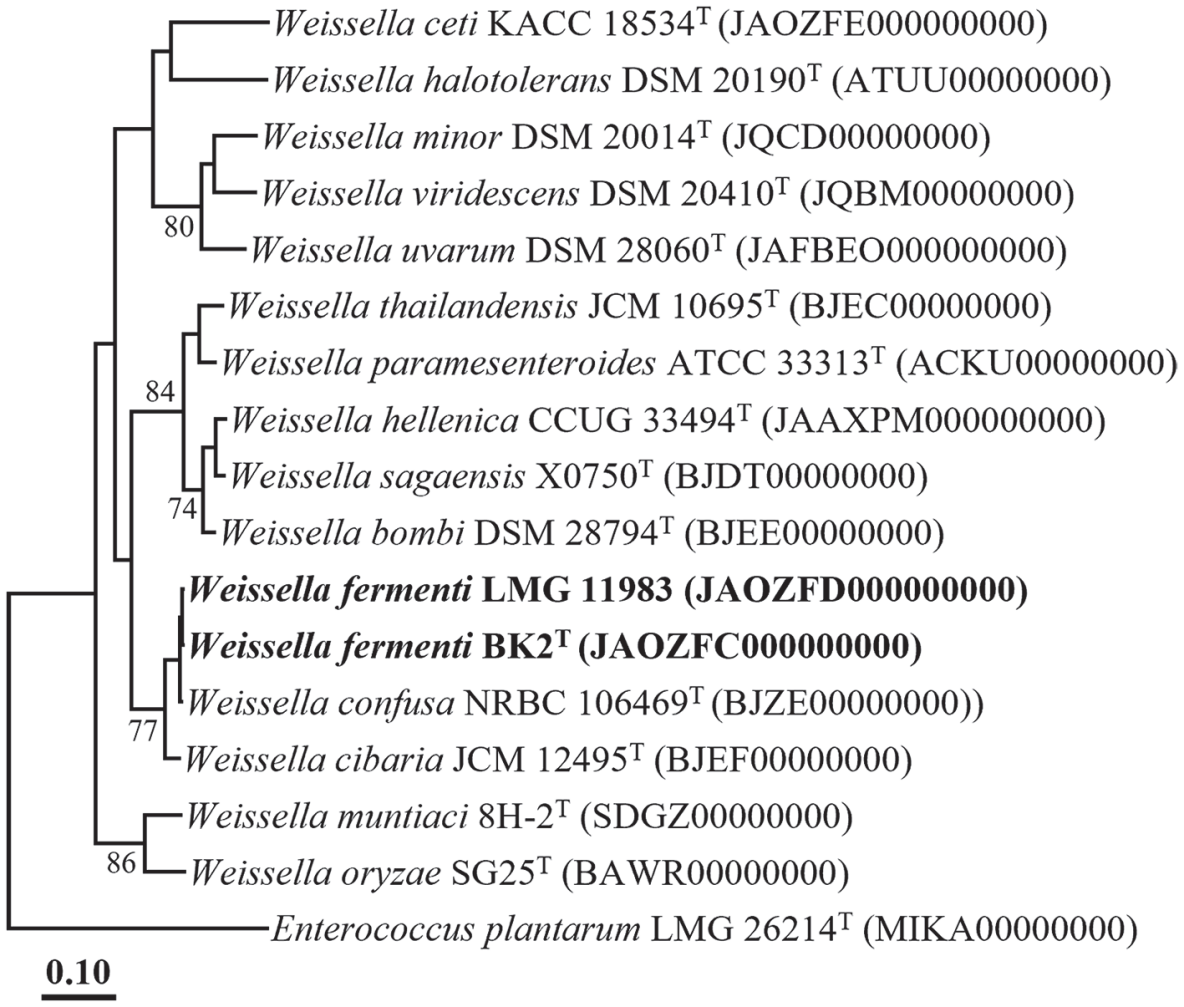
A phylogenomic tree showing the phylogenetic relationships between strains BK2^T^ and LMG 11983 and closely related species, based on the concatenated amino acid sequences of 92 housekeeping core genes. Bootstrap values (based on 1,000 replications) exceeding 70% are indicated at branch points. *Enterococcus plantarum* CCM 7889^T^ (MT758013) was used as the outgroup. The scale bars indicate 0.1 changes per amino acid.

The ANI and dDDH values between strain BK2^T^ and strain LMG 11983 were determined to be 99.9% and 99.7%, respectively, which surpass the established thresholds (ANI ~95%; dDDH 70%) for prokaryotic species delineation [42]. This indicates that strains BK2^T^ and LMG 11983 belong to the same species. Furthermore, the ANI and dDDH values between strain BK2^T^ and closely related reference type strains, including *W. confusa* NRBC 106469^T^, *W. cibaria* JCM 12495^T^, *W. muntiaci* 8H-2^T^, and *W. viridescens* DSM 20410^T^, were determined to be 92.3% and 48.1%, 78.4% and 23.5%, 70.5% and 23.6%, and 70.8% and 22.6%, respectively (Table 1). These results indicate that BK2^T^ and LMG 11983 are strains of a distinct species from other members of the genus *Weissella*. Additionally, the phylogenomic tree constructed based on 92 housekeeping core genes further supports the notion that strains BK2^T^ and LMG 11983 form a distinct phylogenetic lineage within the genus *Weissella* (Fig. 2). Taken together, the genomic relatedness and phylogenomic analyses of strains BK2^T^ and LMG 11983 strongly suggest that they represent a novel species of the genus *Weissella*.

### Phenotypic, Physiological, and Biochemical Characteristics

Strains BK2^T^ and LMG 11983 exhibited robust growth on MRS agar and demonstrated relatively favorable growth on LB agar and TSA, but displayed slow growth on R2A agar, NA, and MA. After 2 days of incubation at 30°C, both strains formed ivory-colored colonies on MRS agar, a characteristic that distinguishes them from closely related reference strains, which formed white colonies (Table 2). The cells of strains BK2^T^ and LMG 11983 were observed to be Gram-stain positive, non-motile rods with dimensions of 0.7–0.8 μm in width and 2.0–2.2 μm in length (Fig. S2). These strains produced both the D- and L-enantiomers of lactic acid in an approximate ratio of 85:15, similar to the other reference strains, except *W. muntiaci* NBRC 113537^T^, which exclusively produced D-lactic acid (Table 2). This result aligns with the *ldh* gene types present in strain BK2^T^, *W. confusa* NBRC 106469^T^, and *W. cibaria* JCM 12495^T^ and their reference strains (Table 1).

Several phenotypic properties were found to be shared between strains BK2^T^ and LMG 11983 and their closely related *Weissella* species. These properties included production of acetoin, activity of catalase, oxidase, acid phosphatase, naphthol-AS-BI-phosphohydrolase, and tryptophan deaminase, fermentation OF D-glucose, and acid production from D-ribose, D-xylose, D-glucose, D-fructose, D-mannose, gluconate, esculin, and *N*-acetyl-glucosamine. However, strains BK2^T^ and LMG 11983 can be differentiated from their closely related *Weissella* species based on several other phenotypic properties, such as colony color and fermentation of L-arabinose, D-mannitol, and D-maltose, as described in Table 2. On the other hand, the phenotypic characteristics presented in Table 2 demonstrated that strains BK2^T^ and LMG 11983 shared nearly identical properties, providing further evidence for their classification as different strains of the same species.

The ability of strain BK2^T^ to thrive specifically in kimchi fermentation cannot be fully explained by genomic analysis or the examination of several metabolic and physiological characteristics alone. However, it is noteworthy that strain LMG 11983, belonging to the same species as strain BK2^T^, was isolated from grass silage, an environment similar to kimchi in terms of plant fermentation. Similarly, *W. confusa* and *W. cibaria*, which are closely related to strains BK2^T^ and LMG 11983, have been isolated from sugar cane and chili bo (a Malaysian food ingredient), respectively, which are also vegetable fermentation environments. These observations suggest that strains BK2^T^ and LMG 11983, as well as *W. confusa* and *W. cibaria*, may have successfully adapted to plant fermentation environments. By contrast, *W. muntiaci* and *W. viridescens*, which are phylogenetically distant, have been isolated from fecal and meat fermentation environments, respectively. Considering that the genomes of strains BK2^T^ and LMG 11983 are larger than those of *W. confusa* and *W. cibaria*, it is plausible that strains BK2^T^ and LMG 11983 may have an advantage in adapting to complex environments, such as kimchi or grass silage, compared to *W. confusa* and *W. cibaria* isolated from simple sugar environments, such as sugar cane or chili bo.

### Chemotaxonomic Characteristics

Strains BK2^T^ and LMG 11983 exhibited appreciable amounts (>10% of total fatty acids) of C_18:1_ *ω*9*c*, C_16:0_, and summed feature 7 (consisting of C_19:1_ *ω*6*c* and/or C_19:1_ *ω*7*c*) as major cellular fatty acids. The overall fatty acid profiles of strains BK2^T^ and LMG 11983 were nearly identical to closely related *Weissella* reference strains, particularly *W. confusa* and *W. cibaria* KACC 11862^T^, albeit with some differences in fatty acid composition (Table 3). PG, two unidentified phospholipids, an unidentified aminolipid, and six unidentified lipids were identified as the major polar lipids in both strains BK2^T^ and LMG 11983 (Fig. S3). The presence of PG in strains BK2^T^ and LMG 11983 aligned with its occurrence in other *Weissella* species, while the absence of DPG distinguished strains BK2^T^ and LMG 11983 from other *Weissella* species [9, 13].

In conclusion, based on the phylogenetic analysis, genome relatedness, and phenotypic and chemotaxonomic features, it is evident that strains BK2^T^ and LMG 11983 represent a single novel species of the genus *Weissella*, for which the name *Weissella fermenti* sp. nov. is proposed.

### Description of *Weissella fermenti* sp. nov.

*Weissella fermenti* (fer.men'ti. L. neut. gen. n. *fermenti*, of a fermentation process).

Cells are Gram-stain-positive, facultatively anaerobic, and non-motile short rods (0.7–0.8 μm in width and 2.0–2.2 μm in length). Colonies on MRS agar are ivory-colored, circular, smooth, and convex. Growth occurs at 15–45°C (optimum, 30°C), pH 5.0–8.0 (optimum, 7.0), and 0–3.0% NaCl (optimum, 1.0% NaCl). Catalase- and oxidase-negative. Acetoin is produced, but not indole and H_2_S. Esculin is hydrolyzed, but not tyrosine, casein, gelatin, Tween 20, Tween 80, and starch. Positive for alkaline phosphatase, leucine arylamidase, *α*-chymotrypsin, *β*-glucuronidase, *β*-glucosidase, *β*-galactosidase, valine arylamidase, acid phosphatase, naphthol-AS-BI-phosphohydrolase, and tryptophan deaminase activities, but negative for lysine decarboxylase, ornithine decarboxylase, urease, gelatinase, esterase (C4), esterase lipase (C8), lipase (C14), crystine arylamidase, trypsin, *α*-galactosidase, *α*-glucosidase, *α*-mannosidase, *α*-fucosidase, *N*-acetyl-*β*-D-glucosaminidase, and arginine dihydrolase activities. Citrate is not utilized. Amygdalin, sucrose, D-glucose, and D-mannitol are fermented, but L-arabinose, L-rhamnose, D-melibiose, inositol, and D-sorbitol are not. Acid production is positive from arbutin, amygdalin, cellobiose, gentiobiose, salicin, sucrose, trehalose, D-galactose, D-ribose, D-xylose, D-glucose, D-fructose, D-mannitol, D-mannose, gluconate, esculin, and *N*-acetyl-glucosamine, but negative from L-rhamnose, D-melibiose, 2-keto-gluconate, maltose, inositol, glycerol, erythritol, D-arabinose, D-adonitol, D-lactose, D-melezitose, D-raffinose, D-turanose, D-lyxose, D-tagatose, D-fucose, D-arabitol, L-xylose, L-sorbose, L-fucose, L-arabitol, dulcitol, sorbitol, inulin, starch, glycogen, xylitol, methyl *β*-D-xylopyranoside, methyl *α*-D-mannoside, methyl *α*-D-glucoside, and 5-keto-gluconate. Produces both D-lactate and L-lactate. The major cellular fatty acids (>10%) are C_18:1_ *ω*9*c*, C_16:0_, and summed feature 7 (comprising C_19:1_ *ω*6*c* and/or C_19:1_ *ω*7*c*). PG, two unidentified phospholipids, one unidentified aminolipid, and six unidentified lipids are identified as polar lipids. The DNA G+C content calculated from the whole genome sequence of the type strain is 44.7 mol%.

The type of strain is BK2^T^ (=KACC 22833^T^=JCM 35750^T^), isolated from kimchi in South Korea. The GenBank accession numbers of the 16S rRNA gene and genome sequences of strain BK2^T^ are OP376746 and JAOZFC000000000, respectively.

## Supplemental Materials

Supplementary data for this paper are available on-line only at http://jmb.or.kr.



## Figures and Tables

**Table 1:** General genomic features of strains BK2^T^ and LMG 11983 and related *Weissella* type strains and their genome relatedness.

Characteristic	1	2	3		4	5	6
Genomic features*							
Genome status	Draft	Draft	Draft		Draft	Draft	Draft
No. of contigs	5	2	41		25	41	8
Genome size (Mb)	2.50	2.51	2.19		2.32	2.29	1.54
G+C content (mol%)	44.7	44.7	44.7		45.1	40.4	40.8
No. of total genes	2,433	2,506	2,121		2,198	2,345	1,546
No. of protein-coding genes	2,299	2,275	2,050		2,090	2,243	1,475
Proteins assigned to COG (%)	1,426 (62.0)	1,411 (62.0)	1,431 (69.8)		1,491 (71.3)	1,384 (61.7)	1,149 (77.9)
No. of rRNAs (5S, 16S, and 23S)	9, 8, 8	9, 8, 8	1, 1, 1		7, 2, 2	4, 1, 1	1, 0, 1
No. of total tRNA genes	83	84	49		77	74	46
No. of noncoding RNAs	3	3	3		3	3	3
No. of CRISPRs^†^	1	1	–		–	–	–
Lactate dehydrogenase (LDH)^a^							
L-LDH (EC 1.1.1.27)	1	1	1		2	–	1
D- LDH (EC 1.1.1.28)	1	1	1		1	1	1
D- LDH (EC 1.1.5.12)	1	1	1		–	–	1
ANI value (%)†	Taxa			dDDH value (%)^†^			
	1	–	99.7	48.1	23.5	23.6	22.6
	2	99.9	–	48.1	23.5	23.6	22.6
	3	92.3	92.4	–	23.2	20.9	20.1
	4	78.4	78.3	78.5	–	20.2	19.8
	5	70.5	70.3	70.4	70.6	–	20.3
	6	70.8	70.8	70.7	70.7	69.4	–

Taxa: 1, strain BK2^T^ (JAOZFC000000000); 2, strain LMG 11983 (JAOZFD000000000); 3, *W. confusa* NBRC 106469^T^ (BJZE00000000); 4, *W. cibaria* JCM 12495^T^ (BJEF00000000); 5, *W. muntiaci* 8H-2^T^ (SDGZ00000000); 6, *W. viridescens* DSM 20410^T^ (JQBM00000000). *The information was obtained from GenBank and annotated by the NCBI prokaryotic genome annotation pipeline (PGAP; www.ncbi.nlm.nih.gov/genome/annotation_prok/).

^†^CRISPR, clustered regularly interspaced short palindromic repeat; ANI, average nucleotide identity; dDDH, digital DNA-DNA hybridization.

**Table 2:** Differential phenotype characteristics of strains BK2^T^ and LMG 11983 and closely related type strains of the genus *Weissella*.

Characteristic	1	2	3	4	5	6
Isolation source	Kimchi	Grass silage	Sugar cane	Chili bo	Feces	Cured meat
Colony color	Ivory	Ivory	White	White	White	White
Enzyme activity (API ZYM)* of:						
Alkaline phosphatase	+	+	+	+	–	–
Leucine arylamidase	+	+	+	–	+	+
*α*-Chymotrypsin	+	+	+	–	–	–
*β*-Glucuronidase, *β*-glucosidase, valine arylamidase	+	+	–	–	–	–
*α*-Glucosidase	–	–	–	–	–	+
Enzyme activity (API 20E)* of:						
*β*-Galactosidase	+	+	–	–	–	–
Arginine dihydrolase	–	–	+	+	+	–
Citrate utilization (API 20E)	–	–	–	–	+	+
Fermentation (API 20E)* of:						
Amygdalin, sucrose	+	+	+	+	–	–
D-Mannitol	+	+	–	–	–	–
Acid production (API 50 CHL)* from:						
Arbutin, amygdalin, cellobiose, gentiobiose, salicin, D-galactose, sucrose	+	+	+	+	–	–
Trehalose	+	+	–	–	+	+
D-Mannitol	+	+	–	–	–	–
2-Keto-gluconate	–	–	+	–	+	+
Maltose	–	–	+	+	–	–
D-Arabinose	–	–	–	+	+	+
Lactic acid configuration*	d, l	d, l	d, l	d, l	d	d, l

Taxa: 1, strain BK2^T^ (this study); 2, strain LMG 11983 (this study); 3, *W. confusa* KACC 11841^T^ [8]; 4, *W. cibaria* KACC 11862^T^ [9]; 5, *W. muntiaci* NBRC 113537^T^ [14]; 6, *W. viridescens* KACC 11850^T^ [1].

All strains are facultative anaerobic short rods and positive for the following characteristics: acetoin production, activity* of acid phosphatase, naphthol-AS-BI-phosphohydrolase, and tryptophan deaminase, hydrolysis of esculin, D-glucose fermentation*, and acid production* from D-ribose, D-xylose, D-glucose, D-fructose, D-mannose, gluconate, esculin, and *N*-acetyl-Dglucosamine. All strains are negative for the following characteristics: hydrolysis of casein, gelatin, starch, tyrosine, Tween 20, and Tween 80, production* of indole and H_2_S, activity* of catalase, oxidase, lysine decarboxylase, ornithine decarboxylase, urease, gelatinase, esterase (C4), esterase lipase (C8), lipase (C14), crystine arylamidase, trypsin, *α*-galactosidase, *α*- mannosidase, *α*-fucosidase, and *N*-acetyl-*β*-glucosaminidase, fermentation* of inositol, L-rhamnose, D-melibiose, and Dsorbitol, and acid production* from glycerol, erythritol, L-arabinose, D-adonitol, D-lactose, L-rhamnose, D-melibiose, Dmelezitose, D-raffinose, D-turanose, D-lyxose, D-tagatose, D-fucose, D-arabitol, L-xylose, L-sorbose, L-fucose, L-arabitol, dulcitol, inositol, sorbitol, inulin, starch, glycogen, xylitol, methyl *β*-D-xylopyranoside, methyl *α*-D-mannoside, methyl *α*-Dglucoside, and 5-keto-gluconate. Symbols: +, positive; –, negative.

*These analyses were conducted under the same conditions in this study.

**Table 3:** Cellular fatty acid compositions (%) of strains BK2^T^ and LMG 11983 and closely related type strains of the genus *Weissella*

Fatty acid	1	2	3	4	5	6
Saturated:						
C_14:0_	0.9	1.0	1.1	1.4	7.7	–
C_16:0_	**23.4**	**22.5**	**20.8**	**21.3**	**24.1**	**17.9**
C_17:0_	tr	–	–	–	–	–
C_18:0_	3.2	2.5	3.1	3.4	2.9	2.3
C_20:0_	–	–	–	0.8	3.5	–
Unsaturated:						
C_18:1_ *ω*9*c*	**29.5**	**25.8**	**34.4**	**39.4**	**19.1**	**68.0**
Branched:						
iso-C_19:0_	1.4	1.2	1.8	2.1	1.6	2.9
Hydroxy:						
C_17:0_ 2-OH	1.5	1.5	1.5	2.1	1.7	2.3
Summed feature*:						
3	3.9	5.4	6.1	3.9	5.9	1.3
7	**29.7**	**32.9**	**16.6**	**20.7**	**29.6**	–
8	6.1	7.4	**14.8**	5.0	3.6	4.5

Taxa: 1, strain BK2^T^; 2, strain LMG 11983; 3, *W. confusa* KACC 11841^T^; 4, *W. cibaria* KACC 11862^T^; 5, *W. muntiaci* NBRC 113537^T^; 6, *W. viridescens* KACC 11850^T^. Fatty acids amounting to less than 0.5% in all strains are not shown. Major fatty acids (>10.0%) are denoted in bold; tr, trace amount (< 0.5%); –, not detected. All data were obtained in this study.

*Summed features are fatty acids that cannot be resolved reliably from another fatty acid using the chromatographic conditions chosen. The MIDI system groups these fatty acids together as one feature with a single percentage of the total. Summed features 3, 7, and 8 contain C_16:1_ *ω*6*c* and/or C_16:1_ *ω*7*c*, C_19:1_ *ω*6*c* and/or C_19:1_ *ω*7*c*, and C_18:1_ *ω*6*c*, respectively.
