# Supplementary material for: Description and Genomic Characteristics of Weissella fermenti sp. nov., Isolated from Kimchi
Source: J Microbiol Biotechnol. 2023 Jul 25;33(11):1448–56. doi: 10.4014/jmb.2306.06010 (PMC10699271; doi:10.4014/jmb.2306.06010)
Supplement: Supplementary file 1 [file jmb-33-11-1448-supple.pdf]

J. Microbiol. Biotechnol.
https://doi.org/10.4014/jmb.2306.05010

Supplementary Figures

Weissella fermenti sp. nov., isolated from kimchi and its genomic and metabolic feature

Jae Kyeong Lee[1†], Ju Hye Baek[1†], Dong Min Han[1], Se Hee Lee[2], So Young Kim[3], and Che Ok Jeon[1*]

[1]*Department of Life Science, Chung-Ang University, Seoul 06974, Republic of Korea*
[2]*Microbiology and Functionality Research Group, World Institute of Kimchi, Gwangju, 61755, Republic of Korea*
[3]*Department of Agro-Food Resources, National Institute of Agricultural Sciences, Rural Development Administration, Wanju 55365, Republic of Korea*

*Author for correspondence: Che Ok Jeon (cojeon@cau.ac.kr)

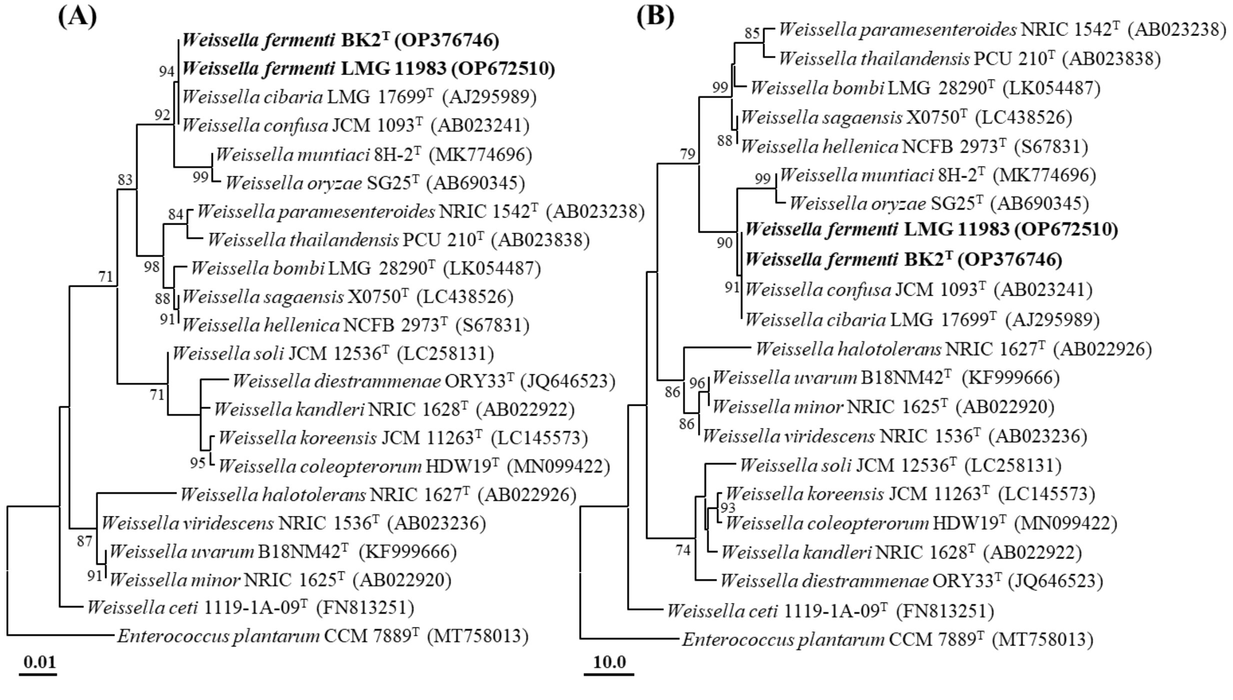

Fig. S1. Maximum likelihood (A) and maximum parsimony (B) trees showing the phylogenetic relationships between strains BK2^T and LMG 11983 and closely related species, based on 16S rRNA gene sequences. Bootstrap values (based on 1000 replications) exceeding 70% are indicated at branch points. *Enterococcus plantarum* CCM 7889^T (MT758013) was employed as the outgroup. The scale bars in panels A and B correspond to substitutions per nucleotide position and the total number of nucleotide substitutions across the entire sequence, respectively.

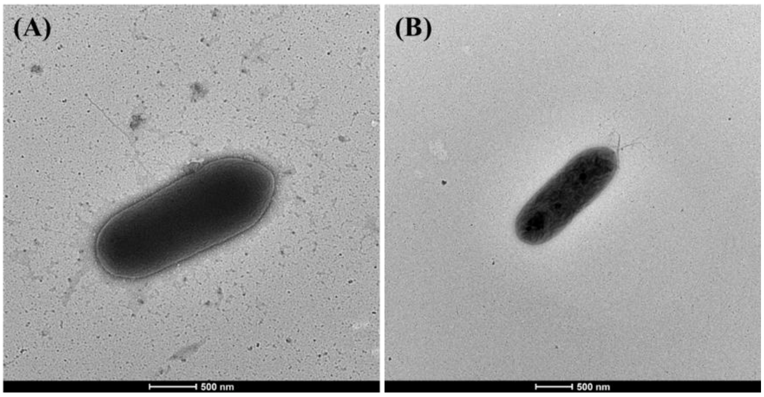

Fig. S2. Transmission electron micrographs showing the general morphologies of negatively stained cells of strains BK2^T (A) and LMG 11983 (B) grown on MRS agar at 30°C for 2 d. Scale bars, 500 nm.

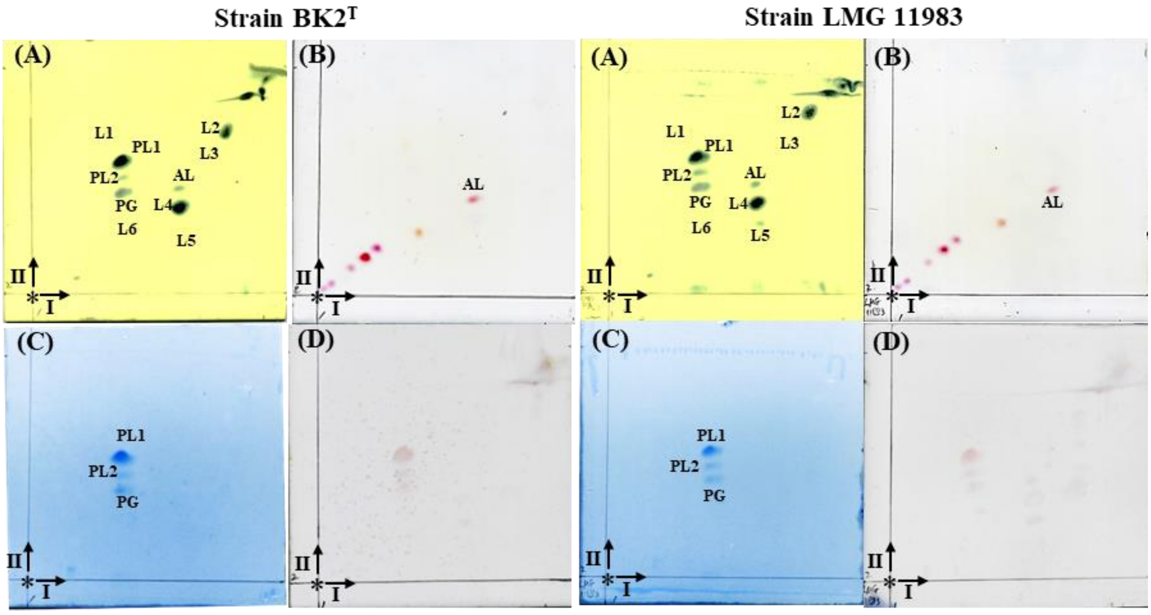

Fig. S3. Two-dimensional thin-layer chromatograms (TLC) showing the polar lipid profiles of strains BK2^T and LMG 11983. Solvent systems: (I) chloroform-methanol-water (65:25:4, v/v/v); (II) chloroform-acetic acid methanol-water (80:15:12:4, v/v/v/v). The TLC plates were sprayed with 10% ethanolic molybdatophosphoric acid, ninhydrin, Dittmer-Lester, α-naphthol, and Dragendorff's reagents for the detection of total polar lipids (A), aminolipids (B), phospholipids (C), and glycolipids (D), respectively. Abbreviations: PG, phosphatidylglycerol; PL, unidentified phospholipids; unidentified aminolipids; L; unidentified lipids.
